# Tuberculosis in renal transplant recipients

**DOI:** 10.4103/0970-1591.42625

**Published:** 2008

**Authors:** Madhivanan Sundaram, Samiran Das Adhikary, George T. John, Nitin S. Kekre

**Affiliations:** Department of Nephrology, Christian Medical College, Vellore, Tamil Nadu, India; 1 Department of Urology, Christian Medical College, Vellore, Tamil Nadu, India

**Keywords:** Organ transplantation, post transplant tuberculosis, tuberculosis in solid organ transplantation, tuberculosis

## Abstract

Infective complications are common after renal transplantation. Tuberculosis (TB) is one of the leading infections following renal transplantation. Reactivation is the most common mode of infection. The factors responsible for this reactivation are chronic liver disease, other coexisting infections, particularly deep mycoses, pneumocystis pneumonia, nocardia, and CMV infections. Cyclosporine use advances the onset of TB to an earlier date. The median onset following transplantation is estimated to be 26 months for those who receive azathioprine and prednisolone as immunosuppression and 11 months for those who receive cyclosporine along with other immunosuppressive agents. Lung is the major site of involvement. Pyrexia of unknown origin is another common presentation. Culture and sensitivity has to be done in all possible cases. Amongst the serological techniques, Interferon alpha production is emerging as the most important. Rifampicin has to be avoided in allograft recipients as it activates cytochrome-P450 enzymes and thereby decreases the therapeutic levels of cyclosporine and prednisolone. The duration of treatment is usually extended for 18 months followed by secondary prophylaxis with isoniazid. Adverse effects of drugs are more often reported in organ recipients and have to be monitored for. Drug resistance is emerging as a problem and appropriate changes in the management have to be carried out.

## INTRODUCTION

Tuberculosis (TB) is a common bacterial infection with high prevalence in developing countries. Recipients of solid organs are more vulnerable than the general population to acquire tuberculosis. Besides, other infections like cytomegalovirus downgrade host immunity and can activate occult TB infection. Tuberculosis causes significant morbidity imposing significant economic burden on the patient and the healthcare agencies. Also, the emergence of primary and secondary drug-resistant *Mycobacterium* may have a severe impact in the organ transplant setting. In this article, we shall review the existing knowledge on post renal transplant TB, the difficulties in diagnosis, changes in the protocols of treatment as compared to the general population, the prognosis, risk factors for acquiring the disease and the current advances. The key words used for search included - TB and organ transplantation, post transplant TB, TB in solid organ transplantation.

## EPIDEMIOLOGY

The incidence and prevalence of TB in India is one of the highest in the world. India contributes 20% of the global incidence of TB which is about 1.8 million new cases of TB in the general population.[1] The incidence in patients on maintenance dialysis is 8.7% and that in renal allograft recipients is 12.3%.[2] The reported incidence from other centers across the country is about the same.[3]

The reported prevalence of post transplant TB is 3.1 to 15% in Asia, 1.5 to 8.5% in South Africa, 1.5 to 3.5% in the Middle East, 1.7 to 5% in Europe and 1.5% in the United States.[4] The actual burden may be much higher in the developing countries. Underreporting may be due to poor maintenance of records and follow-ups.

Both renal and other solid organ transplant recipients are at a higher risk. Thus, the incidence and prevalence is several fold higher in the transplant population as compared to the general population.

## TIME OF ONSET

About 45-60% of TB occurs in the first year after transplantation. A global review on TB estimated the median time for onset at nine months post transplantation.[4] John *et al.*, have shown the median onset to be 26 months for those who received azathioprine and prednisolone as immunosuppression and 11 months for those who received cyclosporine along with other immunosuppressive agents.[5] An earlier occurrence was noted with non-renal solid organ transplantation, cyclosporine, anti CD3 therapy, malnutrition secondary to prolonged dialysis, relative immunodeficiency and exposure to the organism in hospital setting.[6] Data on the role of newer immunosuppressives is emerging. Immunosuppression with tacrolimus or mycophenolate has also been associated with the development of TB earlier in the post transplantation period and also in younger patients.[7] Tailored immunosuppression, with stringent monitoring of the drug levels is expected to decrease the incidence and prevalence in the future.

## RISK FACTORS FOR POST TRANSPLANT TUBERCULOSIS

The major risk factors for TB are chronic liver disease (2 times), other coexisting infections, particularly deep mycoses, pneumocystis pneumonia and nocardia (1.6 times), OKT3 (1.8 times), and CMV infections (2.25 times). Disseminated disease may also be more common with OKT3 and CMV.[8] Cyclosporine use advances the onset to an earlier date and patients on cyclosporine seldom develop TB later than six years.[8] The factors associated with death on univariate analysis were the recipient age, HLA ≤1 antigen match, prednisolone - azathioprine immunosuppression, pre-transplantation TB, post-transplant TB (after two years), chronic liver disease (>6 years), diabetes mellitus, post-transplant diabetes mellitus (>5 years), and the presence of other coexisting infections.[8] HLA A68 (28)/A69 (28) locus appeared to predispose toward post-transplantation TB in the Indian population.[9] Case reports are available where TB manifests immediately after substituting azathioprine with mycophenolate.[10]

## NATURAL HISTORY OF TUBERCULOSIS

As aerosol contributes to the dissemination of the bacilli, the lung is the most common route of acquiring the infection. Inhalation of the organism leads to one of the four possible outcomes- *immediate clearance, chronic or latent infection, rapidly progressive disease, activation after many years*.

Following inhalation, the innate immune system phagocytoses the bacilli and attempts to kill the organism. If this does not happen, the cytokines released in that attempt attract inflammatory cells and promote formation of a granuloma. Failure in containing the infection at this site leads to spread to local lymph nodes and produces regional adenopathy. This parenchymal involvement and the regional adenopathy is termed Ghon complex. Effective cell-mediated immunity is established in about six weeks and this contains further progression of the infection. Any failure by the host to mount an effective cell-mediated immune response and tissue repair leads to progressive destruction of the lung. Erosion of blood vessels and hematogenous spread to other organs leads to disseminated tuberculosis.

Uncontained disease may undergo reactivation when there is proliferation of the organism. This usually occurs when the host's immunity is suddenly compromised. The immunosuppressive conditions that lead to reactivation are HIV infection, end stage renal disease, corticosteroid use, diabetes mellitus, malignancy, old age and malnutrition. The reactivated disease is usually confined to one organ and the lung is the most common site involved, particularly the upper lobe.

## NATURAL HISTORY IN ORGAN RECIPIENTS

Reactivation from previously acquired infection is the predominant mode of developing Tuberculosis in developing nations. Re-infection occurs only in a minority. This should especially be suspected in the presence of multidrug-resistant organisms. The engrafted organ has also been shown to be the carrier in some rare cases. Finger printing with restriction fragment length polymorphism may help in tracking transmission in areas of low endemicity and also nosocomial spread of TB in transplant units.

## CLINICAL MANIFESTATIONS

**Figure 1:**
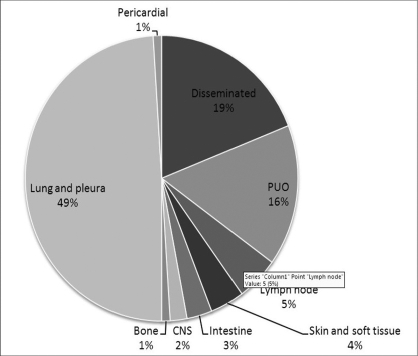
Location of post-transplant tuberculosis

The location of post-transplantation tuberculosis is given in Figure 1. This is based on an analysis of 166 patients with tuberculosis. The lung is the major site of involvement. The other characteristic manifestation is pyrexia of unknown origin. If there is no other conclusive evidence for other infections, empirical anti-tuberculous therapy is usually resorted to.[8]

### Pulmonary tuberculosis

Classical manifestations of the respiratory system like cough, hemoptysis, and shortness of breath are nonspecific and may indicate a variety of infective, immunological, non-immunological pathology in the post-transplant setting. Tuberculosis should be suspected in any patient with radiological evidence of pulmonary infiltrates. Many a time the diagnosis is made on an otherwise asymptomatic patient who undergoes a chest X-ray for other reasons. Cavity, a frequent radiological feature in immunocompetent pulmonary TB, is uncommon in allograft recipients. Fibrosis, regional adenopathy and pleural effusion may be noted. The upper lobe need not be always involved and the disease may be bilateral. Use of Sirolimus may result in lesser incidence of fibrosis because of the drug's effects on fibrogenic growth factors.

### Extra-pulmonary tuberculosis

#### Disseminated disease

The involvement of the liver, bone marrow, meninges, pericardial disease or milliary disease is considered disseminated disease without burden of proof of a second organ involvement. Besides, involvement of lung with an indirect evidence of another organ is also considered to be supportive of disseminated involvement. Lung involvement is universal in disseminated disease (noted in 85.2% of patients).[6]

#### Allograft tuberculosis

Allograft biopsies done for unexplained graft dysfunction may show granulomas on rare occasions in disseminated tuberculosis. Rarely, granulomas may be visualized in the protocol biopsies performed at the time of transplantation. Lymph nodes in the donor detected at the time of nephrectomy, should be excised for biopsy. Surveillance is advised in patients who receive their graft from donors with risk factors to acquire tuberculosis.

#### Genitourinary tuberculosis in allograft recipients

Though rare, case reports are available on genitourinary TB in allografts. The presentation is usually cryptic with constitutional symptoms and graft dysfunction resulting from rejections.[11] The outcome of treatment is usually good.[12]

## DIAGNOSIS

### Microbiological diagnosis

- Acid-fast staining to demonstrate the bacilli in sputum is the most frequently employed method for pulmonary tuberculosis. However, if expectoration is scanty or if there is an unexplained fever or abnormal chest radiograph, gastric juice aspiration may be used. This method has higher sensitivity and reduces the chances of nosocomial exposure to healthcare personnel that otherwise is the case in sputum collection, induction of sputum and bronchoscopy.[13] Acid-fast staining may also be employed in tissue specimens obtained from lymph nodes, bone marrow specimens, liver, kidney, pleural and peritoneal samples. Auramine-rhodamine or auramine O fluorescence staining, permits rapid scanning of the slides and is more sensitive (requires approximately 10^ 4^ organisms per ml sputum for visualization) than the older Ziehl-Nielsen method.
- Culture and sensitivity has to be done in all possible specimens as drug resistance is being increasingly reported. However, it may not help in the immediate diagnosis as it usually takes up to six weeks to obtain a positive report in solid media like Lowenstein Jensen. It may take an additional two weeks for the sensitivity to be reported. Radiolabelled nutrient substrate utilization, gas pressure monitoring and fluorescence emission are used in diagnosis in liquid media (e.g. BACTEC). These methods help in earlier detection (two weeks) of growth.

### Imaging methods

High-resolution computerized tomography, chest tomograms with characteristic lesions may help in improving diagnosis.

Positron emission tomography has also been found useful in diagnosis.[14]

### Molecular techniques

Polymerase chain reaction can amplify genetic material from specimens and can be detected using DNA probes. These tests have specificity close to 100% but the sensitivity is variable.[15]

### Histopathology

Granuloma with caseation is the histological hallmark. Any histopathological tissue with evidence of granuloma should be suspected to be due to tuberculosis unless proved otherwise.

### Serological techniques

Detection of antibodies IgG and IgM have no role in diagnosis.

### Purified protein derivative test

Purified protein derivative (Ppd) test is used for detecting latent tuberculous infection. It depends on Type IV immune response. As there are issues in the administration of the agent, interpreting the test, particularly in those who have received BCG vaccination and in asking the patient to come for a revisit to assess the response, other methods are being increasingly utilized. Besides, the test lacks specificity in developing countries. Therefore, this test is not useful in either screening or diagnosis of tuberculosis.

### Interferon gamma detection (IF γ) detection

T cells of patients infected with tubercle bacilli respond by increased production of IF γ. High level of IF γ indicative of TB infection. Interferon gamma response is very specific to Mycobacterium tuberculosis, as RD1 antigens are not shared by other non pathological tuberculous species or BCG. An ELISA (Quantiferon gold) and an immunospot assay (Ellispot) are used for assessing the interferon γ production. They offer high specificity in detecting infection in immunosuppressed patients. The validity of this test in regions with high exposure to tuberculosis like India and its role in immunosuppressed hosts like solid organ recipients need further evaluation.

### Tuberculosis in dialysis patients

Ten percent of ESRD patients were diagnosed to have have pre transplant tuberculosis[6]. This is secondary to the disruption of cell mediated immune response responsible for killing of intracellular Mycobacterium. The common sites of involvement are lung, lymphnode, pleura and peritoneum PUO is a frequent mode of presentation.[16] The risk factors proposed are advanced age, unemployment, reduced body mass index, decreased serum albumin, hemodialysis, Asian and Native American race, ischemic heart disease, smoking, illicit drug use and anemia.[17] One other study noted hypercalcemia and reversed Globulin/albumin ratio in these patients.[18] Treatment for those not planning for transplantation is short-course chemotherapy with a four-drug regimen. Death was not directly associated with TB infection.[18]

### Drug-resistant tuberculosis

Primary drug resistance is increasingly being reported in the general population. Resistance to isoniazid, rifampicin, pyrazinamide, streptomycin and ofloxacin has been reported. Resistance to ethambutol is uncommon. Primary multidrug resistance has been reported in our center. Secondary resistance due to poor compliance is increasingly encountered.

## TREATMENT OF TUBERCULOSIS

Treatment of TB requires a multidrug regimen administered for appropriate duration in adequate doses. Compliance is equally important. The antituberculous drugs in common use are isoniazid, rifampicin, pyrazinamide and ethambutol and one of the quinolones. The first three are referred to as the first-line essential drugs and are the mainstays of the short-course therapy. The last two are the first-line supplemental agents. The second-line agents have severe toxicity and are used only when there is resistance to first-line agents.

Isoniazid, ethambutol, pyrazinamide are safe agents. Rifampicin, a vital drug in the ATT regimen induces cytochrome-c P450 microsomal enzyme system which is responsible for metabolizing cyclosporine, sirolimus and prednisolone. This unpredictable interaction has led to acute rejections in 30% and graft loss is 20% and hence rifampicin should be avoided in solid organ transplantation.[4] The dose of calcineurin inhibitors may have to be increased two- to fivefold to overcome this effect.[19] Ofloxacin, a quinolone with antimycobacterial property is usually added to make up the four-drug regimen. Though not approved in some countries for this indication, it is considered as effective as ethambutol and its adverse effects are not significant.

Patients on ATT should be monitored for side-effects, which may be more common than the general population because of the use of other therapeutic agents. The major ones of INH are peripheral neuropathy, liver dysfunction and rarely, psychosis. The first of these can be prevented by supplementing pyridoxine. The dose of pyridoxine can be increased if the patient develops symptoms despite being on pyridoxine. If peripheral neuropathy continues to persist, the INH dose needs to be dropped or sometimes withdrawn. The incidence of hepatotoxicity is higher when INH is used. This happens even in patients without chronic liver disease due to hepatotrophic virus infections. The side-effect is, however, not serious. Transient elevation of AST is noted without evidence of jaundice. Hepatotoxicity resulted in withdrawal of INH in 2.5%.[4] Pyrazinamide is also hepatotoxic and ocular toxicity is a side-effect of ethambutol. CMV retinitis should be differentiated. Periodic eye evaluation is necessary while on drugs. Hyperuricemia, a common side-effect with pyrazinamide and ethambutol is aggravated in the presence of cyclosporine. Allopurinol potentiates the marrow toxicity of azathioprine and hence cannot be used to control hyperuricemia.

**Duration of therapy:** The regimens used in different centers vary in dose and duration. These regimens are not validated and are based on individual preferences and response. Randomized studies in this area are required. We use a four-drug regimen: pyrazinamide (three months); ofloxacin (nine months); INH and ethambutol (18 months). The dose of INH and ethambutol has to be adjusted for the degree of renal function. If rifampicin has to be used for those who are not on cyclosporine, prednisolone dose has to be doubled.

### Chemoprophylaxis

In endemic areas, primary prophylaxis is necessary to prevent infections, particularly for those who receive organs from donors suspected or treated for TB and secondary prophylaxis after treatment of pre-transplant tuberculosis. Indications like positive skin sensitivity do not apply in developing countries.

## ATYPICAL MYCOBACTERIAL INFECTIONS

Infections with atypical mycobacteria are infrequent in renal transplant recipients but cause serious morbidity. We have reported four cases in the period between 1997 and 2003.[18] *M. chelonei, M. fortuitum, M. abcessus* and *M. terrae* have been seen in these patients. *M. kansasii, M. avium intracellulare, M. gastri, M. scrofulaceum* and *M. thermoresistible* are the other agents reported.[20] Staining and culture are essential for diagnosis. *In vitro* antibiotic sensitivity is essential for treatment. Besides the conventional agents, drugs like clarithromycin, azithromycin, amikacin, netilmicin may find a use in treating these agents.

## CONCLUSION

Tuberculosis in the allograft recipient is a common problem, particularly in developing countries where the incidence and prevalence in the general population is high. This risk is also compounded by the risk factors that exist in these people. The presentation of the disease differs in solid organ recipients and a high index of suspicion is important in diagnosing the problem. Diagnosis is along conventional lines, though the sensitivity and specificity of the investigations vary. The interactions of the ATT drugs with the immunosuppressive agents have to be considered while prescribing and surveillance for adverse effects has to be done. The duration of treatment has to be prolonged and secondary prophylaxis has to be considered. Drug resistance and atypical mycobacterial infections are emerging problems and should be suspected in the non-responding patients.
